# Comparing surrogate indexes for insulin resistance as predictors of type 2 diabetes

**DOI:** 10.1210/clinem/dgag101

**Published:** 2026-03-10

**Authors:** Laura Vazquez, Elsa Vazquez Arreola, Murugasu Nagul, Jonathan Krakoff, Robert L Hanson

**Affiliations:** Phoenix Epidemiology and Clinical Research Branch, National Institute of Diabetes and Digestive and Kidney Diseases, National Institutes of Health, Phoenix, AZ 85004, USA; Phoenix Epidemiology and Clinical Research Branch, National Institute of Diabetes and Digestive and Kidney Diseases, National Institutes of Health, Phoenix, AZ 85004, USA; Phoenix Epidemiology and Clinical Research Branch, National Institute of Diabetes and Digestive and Kidney Diseases, National Institutes of Health, Phoenix, AZ 85004, USA; Phoenix Epidemiology and Clinical Research Branch, National Institute of Diabetes and Digestive and Kidney Diseases, National Institutes of Health, Phoenix, AZ 85004, USA; Phoenix Epidemiology and Clinical Research Branch, National Institute of Diabetes and Digestive and Kidney Diseases, National Institutes of Health, Phoenix, AZ 85004, USA

**Keywords:** insulin resistance, insulin sensitivity, type 2 diabetes, prediction

## Abstract

**Context:**

Decreased insulin sensitivity (insulin resistance) is associated with increased type 2 diabetes (T2D) risk. Identifying reliable surrogate indexes that use standard clinical assays might improve the prevention of T2D.

**Objective:**

The objective was to compare the ability of 18 surrogate indexes of insulin resistance to predict T2D.

**Design:**

Cohort study of indigenous Americans from the southwest United States.

**Setting:**

Community-based longitudinal study.

**Patients and Other Participants:**

We studied T2D risk in 2260 people followed for up to 14.5 years (509 cases of T2D). We estimated correlation coefficients (*r*) of all indexes with sensitivity (M) measured by hyperinsulinemic–euglycemic clamp in 286 persons. We calculated indexes obtained from oral glucose tolerance tests (OGTTs) using fasting and 2-hour glucose and insulin measures.

**Interventions:**

None.

**Main Outcome Measures:**

Predictive performance was assessed by the hazard ratio per SD and by the area under the receiver operating curve (*AUC*).

**Results:**

Indexes calculated from OGTTs performed best; Matsuda had the highest *r* with M (0.691); Gutt and Cederholm had the highest AUCs (0.728 and 0.728, *P* < .05 compared with all other indexes). In indexes that only used fasting insulin levels, the quantitative insulin sensitivity check index and Homeostatic Model for Insulin Resistance had the best performance with equal *r* (0.644) and AUC (0.701). Among indexes that did not require insulin measurement, the metabolic score for insulin resistance (METS-IR) and single-point insulin sensitivity index had the highest *r* (0.597 and 0.595), while METS-IR had the highest AUC (0.688).

**Conclusion:**

Surrogate measures based on OGTTs are best for predicting T2D, while measures based on fasting insulin perform better than those that do not measure insulin.

Insulin resistance can be characterized as a decreased ability of insulin to dispose of glucose (1). As a result, excess glucose in the bloodstream contributes to the development of type 2 diabetes (T2D) (1-3). Diabetes is associated with long-term complications such as retinopathy, nephropathy, and cardiovascular diseases (1, 4). In the United States, the prevalence of insulin resistance based on the Homeostatic Model for Insulin Resistance (HOMA-IR) was ∼40% in adults according to estimates from the 2007-2008 to 2017-2018 National Health and Nutrition Examination Survey cycles (5). Measuring insulin resistance may help to identify individuals at risk for T2D for early intervention.

The hyperinsulinemic–euglycemic clamp (HEC) is the gold-standard test to measure insulin resistance (6); however, it is invasive, expensive, and laborious (4, 7). It requires trained staff and continuous infusion of insulin and glucose to maintain steady blood glucose levels and to calculate the glucose infusion rate (8). Therefore, several surrogate indexes of insulin resistance that may be more clinically convenient and easier to assess for large-scale studies have been developed (9). Some of these surrogate indexes require an oral glucose tolerance test (OGTT), while others require measurement of serum insulin concentrations in the fasting state only (10). Still, measurement of serum insulin levels is expensive, and the assays are not well-standardized for clinical use. Thus, there is interest in surrogate indexes calculated from measurements of plasma glucose and triglyceride levels to estimate insulin resistance (11, 12). These tests only require 1 blood collection with minimal discomfort to the subjects and are less costly.

The objective of this study was to compare the ability of 18 surrogate indexes of insulin resistance to predict future T2D development in indigenous Americans living in the southwest United States. We also assessed the associations of these 18 surrogate indexes with the gold-standard measure of insulin sensitivity (M), obtained from a HEC performed in some of the subjects.

## Materials and Methods

### Data Collection

Data for the current analyses were obtained from a longitudinal study in an indigenous American community living in the southwestern United States (13). Regardless of health status, members of the community age ≥5 years were invited to participate in research examinations every 2 years. Data collected at these outpatient visits include a medical history, height, weight, and waist circumference. Laboratory studies include plasma glucose and serum insulin concentrations in the fasting state and 2 hours after a 75 g oral glucose load. Hemoglobin A1c (HbA1c) was measured by high-performance liquid chromatography using the Bio-Rad MDMS system from 1989 to 2000. From 2001 until the end of the study in 2007, we used the A1C 2.2 Plus Glycohemoglobin Analyzer (Tosoh Diagnostics, San Francisco, CA, USA). A regression equation was used to convert the older HbA1c assay results to the newer assay. Testing for fasting serum triglyceride was started in 1993 and continued until 2007. Insulin was measured from 1987 to 1997 by a Concept4 analyzer (ICN Radiochemicals, Costa Mesa, CA, USA) and from 1998 to 2007 by an Access analyzer (Beckman Instruments, Fullerton, CA, USA).

We compared the performance of 18 surrogate indexes of insulin resistance to predict the future development of T2D. We employed surrogate indexes that have been widely used in clinical research and epidemiologic studies. Indexes were calculated from measures of insulin and glucose obtained from OGTTs, fasting triglyceride, and high-density lipoprotein (HDL) cholesterol levels. Additional measures include height, weight, body mass index (BMI), and waist circumference. Indexes calculated from OGTT measures were Matsuda, Belfiore, Gutt, Avignon (Sib, Si2h, and SiM), Stumvoll, and Cederholm. For indexes that can use multiple time points in the OGTT, we only used fasting and 2-hour measures because these are widely available in epidemiological studies. Indexes requiring fasting insulin levels but no OGTT include the HOMA-IR, the quantitative insulin sensitivity check index (QUICKI), and McAuley. Plasma fasting insulin level alone was also included as an index. Indexes that do not utilize insulin measures in their calculation were the single-point insulin sensitivity index (SPISE), metabolic score for insulin resistance (METS-IR), triglycerides and HDL cholesterol ratio, triglycerides and glucose index (TyG), triglycerides and glucose ratio (TG/G), and lipid accumulation product. Details for calculation of each index are presented in Table 1.

**Table 1:** Formulas for surrogate indexes of insulin sensitivity/resistance

M (14)	M = GIR − [G_120_,_mg/dL_ − G_90_,_mg/dL_]/30_min_ * V_glucose_,_dL/kg_ − UC
Indexes requiring an OGTT	
Gutt (15)	ISI_0,120_ = [75 000 + (G_0_ − G_120_) * 0.19 * m]/[120 * G_mean_ * log(*I*_mean_)]
Matsuda (16)	ISI_Matsuda_ = 10 000/[(√G_0_) * I_0_ * G_mean_ * *I*_mean_]
Belfiore (17)	ISI_Belfiore_ = 2/[(G_S_/G_N_) * (I_S_/I_N_) + 1]
Avignon (Sib) (18)	Sib = 10^8^/(*I*_0_ * *G*_0_ * *VD*)
Avignon (Si2h) (18)	Si2h = 10^8^/(*I*_120_ * *G*_120_ * *VD*)
Avignon (SiM) (18)	SiM = [(0.137 * Sib) + Si2h]/2
Stumvoll (19)	ISI_Stumvoll_ = 0.156 − 0.0000459 * *I*_120_ − 0.000321 * *I*_0_ − 0.00541 * G_120_
Cederholm (20)	ISI_Cederholm_ = [75 000 + (G_0_ − G_120_) * 1.15 * 180 * 0.19 * m]/[120 * G_mean_ * log(*I*_mean_)]
Indexes requiring fasting insulin	
HOMA-IR (21)	HOMA-IR = (fasting insulin_uIU/mL_ * fasting glucose_mmol/L_)/22.5
QUICKI (22)	QUICKI = 1/[log (fasting insulin_U/mL_) + log (fasting glucose_mg/dL)_]
McAuley (23)	$\left(\frac{\mathrm{MffM}}{I}\right)$ = exp[2.63 − 0.28ln (fasting insulin_μU/mL_) − 0.31ln (fasting triglycerides_mmol/L_)]
Fasting insulin (24)	Fasting insulin_uIU/mL_
Indexes not requiring insulin measures	
SPISE (25)	SPISE = 600 * HDL^0.185^/TG^0.2^/BMI^1.338^
METS-IR (26)	METS-IR = Ln[(2 * *G*_0_)+TG) * BMI)/(Ln(HDL-C)]
LAP (27)	Men: LAP = (Waist_cm_ − 65) * (TG_mmol/L_)
	Women: LAP = (Waist_cm_ − 58) * (TG_mmol/L_)
TyG (28)	TyG = Ln [(fasting TG_mg/dL_ * FPG_mg/dL_)/2]
TG and HDL-C ratio (29)	TG/HDL-C ratio = TG_mg/dL_/HDL-C_mg/dL_
TG/G (30)	TG/G ratio = TG_mg/dL_/FPG_md/dL_

Abbreviations: BMI, body mass index; FPG, fasting plasma glucose; G_0_, fasting plasma glucose concentration; G_90_, plasma glucose concentration in the 90th minute of OGTT; G_120_, plasma glucose concentration in the 120th minute of OGTT; GIR, glucose infusion rate; G_mean_, mean plasma glucose concentration during OGTT; G_S_, G_N_, plasma glucose concentrations expressed as fasting values or as areas obtained during a standard OGTT at 0 and 2 hours; HDL-C, high-density lipoprotein cholesterol; HOMA-IR, Homeostatic Model Assessment of Insulin Resistance; *I*_0_, fasting plasma insulin concentration; *I*_mean_, mean plasma insulin concentration during OGTT; I_S_, I_N_, plasma insulin concentrations expressed as fasting values or as areas obtained during a standard OGTT at 0 and 2 hours; ISI, insulin sensitivity index; LAP, lipid accumulation product; m, body weight (kg); METS-IR, metabolic score for insulin resistance; QUICKI, quantitative insulin sensitivity check index; SPISE, single-point insulin sensitivity index; TG, plasma triglyceride; TG/G, triglycerides and glucose ratio; TyG, triglycerides and glucose index; UC, correction factor for urinary loss of glucose; V, volume of distribution of glucose; VD, glucose distribution volume.

### Inclusion Criteria

This study included participants ≥18 years of age. Participants had at least 1 observation with complete data on measures necessary to calculate all 18 surrogate indexes of insulin resistance, with the baseline examination defined as the first observation with complete data on these measures. We only included participants who did not have diabetes at the time of their baseline examination and who had at least 1 follow-up visit at which diabetes was assessed. T2D was defined using American Diabetes Association criteria as a documented clinical diagnosis, 2-hour glucose after a 75-g oral glucose load (2-hour PG) ≥ 200 mg/dL (≥11.1 mmol/L), fasting plasma glucose (FPG) ≥ 126 mg/dL (≥7.0 mmol/L), or HbA1c ≥ 6.5% (≥48 mmol/mol) (31). There were 2260 individuals who met these criteria; we refer to them as the population study cohort. To assess whether a definition of diabetes including the 2-hour PG concentration favored indexes based on the OGTT, we also performed a sensitivity analysis, where we defined diabetes based on FPG ≥ 126 mg/dL (≥7.0 mmol/L), HbA1c ≥ 6.5% (≥48 mmol/mol), or self-reported history of diabetes drug use; we refer to these participants as the sensitivity analysis cohort, which included 2343 participants.

**Figure 1:**
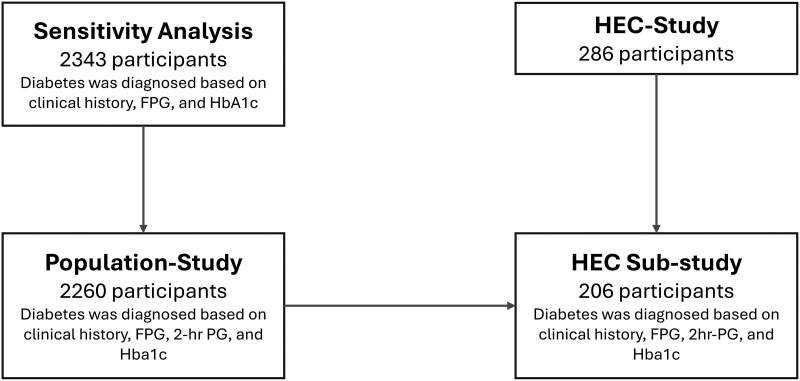
Schema representing the different datasets included in the analyses.

The HEC was performed on a subset of participants who were part of a study designed to determine the action of insulin as previously described (14). We used data from 286 participants of this substudy with HEC measures and data on the surrogate indexes of interest to determine how these indexes were correlated with the gold-standard measure of insulin sensitivity (M). For this cohort, insulin measures were converted across assays by a regression equation to a standard charcoal immunoassay (32). Additionally, we analyzed how M and surrogate indexes compared in the prediction of T2D in 206 of these participants. These participants had follow-up data for future development of T2D, with the same inclusion criteria as those in the population study. We refer to this cohort as the HEC substudy. During the insulin clamp, hepatic glucose production was measured using an infusion of [3-H^3^]-glucose before and at the end of insulin administration as previously described (33). Hepatic insulin resistance was calculated as the reciprocal of the percentage reduction in hepatic glucose production after the insulin infusion compared with baseline. A 25 g IV glucose tolerance test was also conducted in 266 members of the HEC cohort. The acute insulin response (AIR), a measure of insulin secretion, was calculated as the insulin response above the basal level at 3 to 5 minutes after the glucose bolus as previously described (13). Some participants in these studies were also included in a previous analysis of this population that examined a limited number of indexes (13). Studies were approved by the institutional review board of the National Institutes of Health, and all participants provided written informed consent. A schema showing the different studies is shown in Fig. 1.

### Statistical Analyses

Statistical analyses were performed using SAS software (version 9.4; SAS Institute, Cary, NC, USA). Spearman correlation coefficients (*r*) were used to determine the strength of associations of all indexes with M measured by the HEC. Steiger's *Z*-statistic was calculated to test the statistical significance of differences in the correlation with M between pairs of the surrogate indexes (34). To assess associations of indexes with other aspects of glucose metabolism, we also calculated the correlations with hepatic insulin resistance and the partial correlations, with adjustment for M, with AIR. Associations of the indexes with T2D development were assessed separately using Cox proportional hazards regression with adjustment for sex, age, and self-reported fraction of American Indian heritage. The hazard ratio (HR) was expressed per SD of the log-transformed value of each index; to ensure comparability, indexes were standardized to a mean of 0 and SD of 1 within each assay in these analyses. Follow-up years were defined as time from baseline to the first occurrence of diabetes or the last follow-up measurement if the event did not develop. Predictive performance for the development of diabetes was assessed by quantifying the change in the area under the receiver operating curve (AUC) for models containing the different indexes of interest compared with a baseline model that only contained the covariates. AUCs and their SEs were calculated using the methods described by Pencina and D’Agostino (35). The Delong et al method was used to conduct pairwise comparisons of AUCs for the different indexes (36). The incremental improvement in AUC represents the improvement in accuracy for the prediction of the development of diabetes, but it may not fully reflect the ability of a variable (surrogate index) to reclassify risk, particularly when baseline covariates are strongly predictive. We thus additionally calculated the net reclassification improvement (NRI) with respect to the baseline model for each insulin resistance/sensitivity index (37). BMI is strongly associated with both insulin resistance and diabetes risk, and it may be of interest in clinical practice and research studies to assess the effects of insulin resistance independent of BMI. We therefore conducted secondary/exploratory analyses that additionally adjusted for BMI. To assess predictive value beyond that provided by other clinical predictors, we also further adjusted for baseline HbA1c, as well as whether the individual reported that they currently (within the last year) consumed alcohol or smoked cigarettes.

World Health Organization criteria for metabolic syndrome define insulin resistance as belonging to the lowest quartile group of insulin action (eg, as measured by the HEC) (38). In practice, these quartile groups are often defined by the surrogate indexes (39, 40). To assess the diagnostic accuracy for identifying insulin resistance defined by the clamp, we therefore assessed the overlap of the putatively most insulin-resistant quartile group for each index with the most insulin-resistant quartile group defined by the gold-standard clamp measure, and we calculated sensitivity and specificity accordingly.

## Results

Table 2 shows the baseline characteristics for all participants stratified by cohort. The median age and follow-up time for the population study cohort were lower than in the HEC substudy. At least 80% of patients had normal glucose tolerance based on FPG and 2-hour PG. There were 509, 44, and 543 events of diabetes in the population study, the HEC substudy, and the sensitivity analysis cohorts, respectively.

**Table 2:** Baseline demographic and clinical characteristics of participants included in the analysis of future type 2 diabetes development stratified by cohort

	Population Study n = 2260	HEC substudy n = 206	Sensitivity analysis n = 2343
Male, n (%)	900 (39.8)	120 (58.3)	920 (39.3)
Diabetes events, n (%)	509 (22.5)	44 (21.4)	543 (23.2)
Age, years	27.39 (20.49-35.63)	28.44 (23.01-33.15)	27.82 (20.64-36.21)
BMI, kg/m^2^	33.14 (28.4-38.2)	33.07 (28.18-38.53)	33.25 (28.62-38.35)
Waist circumference, cm	104 (94-117)	106.83 (96.01-119.38)	105 (94-117)
Diabetes, follow-up, years	5.59 (3.06-9.14)	7.45 (4.53-10.05)	5.89 (3.27-9.31)
Fasting glucose, mg/dL	91 (85-98)	86.5 (80.5-92.5)	92 (86-99)
2-hour glucose, mg/dL	108 (90-128)	114 (91-145)	109 (90-132)
Cholesterol, mg/dL	172 (152-195)	174.5 (153-195)	172 (152-195)
HbA1c, %	5.2 (4.9-5.5)	—	5.3 (5.0-5.6)
HDL cholesterol, mg/dL	42 (36-51)	44 (38-52)	42 (36-51)
Triglycerides, mg/dL	110 (78.0-156.5)	1.55 (1.13-1.98)	111 (78-158)
Fasting insulin, pmol/L	88.59 (51.84-150.0)	37.89 (28.13-51.87)	90 (52.68-156)
2-hour insulin, pmol/L	456 (193.95-884.97)	157.58 (82.76-274.81)	473.16 (199.74-930)
Insulin sensitivity (M)	—	2.32 (2.04-2.92)	—
Matsuda index	4.01 (2.16-7.82)	1.74 (1.11-2.77)	3.93 (2.07-7.70)
Belfiore index	0.0003 (0.0002-0.0008)	0.005 (0.003-0.011)	0.0003 (0.0001-0.0007)
Gutt index	1.64 (1.27-2.24)	1.33 (1.06-1.72)	1.61 (1.23-2.21)
Avignon index (Si2h)	0.75 (0.34-1.86)	0.33 (0.17-0.74)	0.7 (0.32-1.81)
Avignon index (SiM)	0.74 (0.41-1.52)	0.30 (0.20-0.55)	0.71 (0.39-1.48)
Avignon index (Sib)	4.59 (2.75-7.50)	1.89 (1.37-2.44)	4.51 (2.66-7.37)
Stumvoll index	0.07 (0.03-0.10)	0.12 (0.11-0.13)	0.07 (0.02-0.10)
Cederholm index	28.53 (21.15-40.00)	22.51 (16.75-30.37)	27.880 (20.39-39.61)
HOMA-IR	3.29 (1.89-5.80)	8.19 (5.64-11.85)	3.38 (1.93-6.01)
QUICKI	0.14 (0.13-0.15)	0.12 (0.12-0.13)	0.14 (0.13-0.15)
McAuley index	1.5 (1.24-1.86)	1.06 (0.94-1.27)	1.49 (1.23-1.85)
SPISE	4.26 (3.42-5.42)	4.16 (3.31-5.26)	4.22 (3.40-5.37)
METS-IR	50.83 (42.38-60.11)	50.48 (41.89-61.02)	51.32 (42.60-60.61)
Lipid accumulation product	56.38 (33.66-87.42)	70.05 (41.55-106.82)	57.4 (34.26-88.34)
Triglycerides and HDL cholesterol ratio	2.53 (1.69-3.94)	3.14 (2.00-4.32)	2.56 (1.70-4.03)
Triglycerides and glucose index	8.52 (8.16-8.90)	8.71 (8.29-8.98)	8.53 (8.17-8.92)
Triglycerides and glucose ratio	1.2 (0.85-1.69)	1.58 (1.16-2.03)	1.2 (0.86-1.70)

Data are presented as median (interquartile range) unless otherwise indicated. — indicates not measured.

Abbreviations: BMI, body mass index; HbA1c, hemoglobin A1c; HDL, high-density lipoprotein; HEC, hyperinsulinemic–euglycemic clamp; HOMA-IR, Homeostatic Model Assessment of Insulin Resistance; METS-IR, metabolic score for insulin resistance; QUICKI, quantitative insulin sensitivity check index; SPISE, single-point insulin sensitivity index.

### Correlations with M

Spearman correlation coefficients between M and each surrogate index are shown in Table 3. Indexes using insulin measures generally correlated more strongly with M than those that do not use plasma insulin levels in their calculation. Of all indexes, Matsuda, which requires an OGTT for its calculation, had the highest correlation (0.691) with M, while HOMA-IR and QUICKI had similar values of Spearman correlation with M (*r* = 0.644), which was the highest correlation among indexes that do not require an OGTT. Among the indexes not requiring insulin measures, METS-IR (0.597) and SPISE (0.595) had the highest correlations with M. According to Steiger's *Z*-statistic, Matsuda had a significantly stronger correlation with M than any of the other surrogate indexes. HOMA-IR/QUICKI had a significantly stronger correlation with M than most of the indexes that do not use plasma insulin values (Table 3). With adjustment for M, several indexes (Gutt, Cederholm, Stumvoll, HOMA-IR, QUICKI, fasting insulin, and SPISE) had significant correlations with AIR. Most measures were more weakly correlated with hepatic insulin resistance than with M; the strongest correlations with hepatic insulin sensitivity were observed with SPISE and METS-IR. Correlations among the different indexes are shown in Table S1 (41). These analyses were performed with adjustment only for age and sex. With additional adjustment for BMI, the correlations with M were attenuated, particularly for indexes that include obesity measures (eg, METS-IR, SPISE, lipid accumulation product), but the ranking was generally similar (Table S2) (41).

**Table 3:** Spearman correlation coefficients (*r*) between surrogate indexes and sensitivity (M) measured by HEC in the HEC substudy cohort

Index	*r* with M	HEC n = 286				
		*P* with Matsuda	*P* with HOMA-IR	*P* with METS-IR	*r′* with AIR*^a^*	*r* with HIR
Requiring an OGTT						
Matsuda	0.691	—	.0161	.0084	0.063	0.346*
Belfiore	0.630	<.0001	.3429	.2297	0.0004	0.356*
Gutt	0.556	<.0001	.0157	.1993	−0.166*	0.298*
Avignon (Si2h)	0.478	<.0001	.0002	.0198	−0.069	0.267*
Avignon (SiM)	0.524	<.0001	.0011	.0928	−0.037	0.252*
Avignon (Sib)	0.501	<.0001	<.0001	.0408	0.063	0.141*
Stumvoll	0.442	<.0001	<.0001	.0023	−0.251*	0.215*
Cederholm	0.540	<.0001	.0083	.1214	−0.190*	0.297*
Requiring fasting insulin						
HOMA-IR	0.644*^b^*	0.0161	—	.1163	0.120*	0.271*
QUICKI	0.644	0.0161	—	.1163	0.120*	0.271*
McAuley	0.558	0.0001	.0056	.1761	0.076	0.295*
Fasting insulin	0.642*^b^*	0.0176	.4185	.1324	0.159*	0.285*
Not requiring insulin measures						
SPISE	0.595	0.0081	.1118	.4072	0.125*	0.400*
METS-IR	0.597*^b^*	0.0084	.1163	—	0.114	0.405*
Lipid accumulation product	0.508*^b^*	<.0001	.0021	.0043	0.026	0.343*
Triglycerides and HDL cholesterol ratio	0.355*^b^*	<.0001	<.0001	<.0001	−0.013	0.247*
Triglycerides and glucose index	0.329*^b^*	<.0001	<.0001	<.0001	−0.067	0.214*
Triglycerides and glucose ratio	0.208*^b^*	<.0001	<.0001	<.0001	−0.006	0.166*

The column *r* with M contains the Spearman correlation coefficient of the log-transformed surrogate index with M, the measure of insulin sensitivity from the HEC with adjustment for age and sex. The column *P* with Matsuda contains the *P*-value for Steiger's Z-statistic testing statistically significant differences between the correlations with M of Matsuda index and of each of the other surrogate indexes. Similarly, the columns *P* with Matsuda, *P* with HOMA-IR, and *P* with METS-IR contain *P*-values for Steiger's Z-statistic testing differences between the correlations with M of Matsuda, HOMA-IR, and METS-IR and each of the other surrogate indexes.

*r*′ = Spearman correlation coefficients between surrogate indexes of insulin resistance additionally partialed by M with insulin secretion. AIR had 20 missing values, resulting in partial Spearman correlation coefficients based on 266 observations with all indexes.

*r* with hepatic insulin resistance had 93 missing values resulting in the Spearman partial correlation coefficients based on 193 observations.

^a^AIR is the gold standard measure of insulin secretion from IV glucose tolerance tests.

^*b*^These indexes provide a measure of insulin resistance. Since M provides a measure of insulin sensitivity, we reversed the signs of these indexes so they are in the same direction as M, providing a measure of insulin sensitivity rather than insulin resistance.

^*^ *P*-value ≤ .05.

Abbreviations: AIR, acute insulin response; HEC, hyperinsulinemic–euglycemic clamp; HOMA-IR, Homeostatic Model Assessment of Insulin Resistance; METS-IR, metabolic score for insulin resistance; OGGT, oral glucose tolerance test; QUICKI, quantitative insulin sensitivity check index; SPISE, single-point insulin sensitivity index.

### Diagnosis of Insulin Resistance

Properties of each index for identifying insulin resistance, defined as the lowest quartile group of M, are shown in Table S3 (41). Insulin resistance defined by the lowest quartile group of Matsuda had 57.7% sensitivity and 86.0% specificity for insulin resistance defined by M; this was the optimal combination among all indexes. Defining insulin resistance by quartiles of HOMA-IR had 49.3% sensitivity and 83.3% specificity, while values for METS-IR were 46.5% and 82.3%, respectively.

### Diabetes in the Population Study Cohort

Table 4 shows the associations of surrogate indexes with the risk of T2D for the population study cohort. Indexes requiring an OGTT tended to have stronger HRs for T2D than other measures; among these indexes, Gutt [HR = 2.34, 95% confidence interval (CI): 2.07, 2.64], Cederholm (HR = 2.28, 95% CI: 2.04, 2.55), and Matsuda (HR = 2.11, 95% CI: 1.89, 2.34) had the highest HRs. All indexes except for the TG/G were significantly associated with future T2D development. However, METS-IR had the highest HR among indexes that do not require insulin (HR = 1.80, 95% CI: 1.64, 1.98). QUICKI (HR = 2.04, 95% CI: 1.81, 2.28) had the highest HR among the indexes that require insulin levels followed by HOMA-IR (HR = 1.97, 95% CI: 1.77, 2.19). AUCs for surrogate indexes of insulin resistance for predicting diabetes are shown in Table 4. Indexes requiring an OGTT had the highest AUCs among all indexes, while indexes not requiring insulin measures had the lowest. Cederholm had significantly higher AUCs than all other indexes, except Gutt (*P* < .05). HOMA-IR performed significantly better than fasting insulin and most indexes that do not use insulin concentrations, except SPISE and METS-IR. NRI calculations indicate that Matsuda (NRI = 0.613), Stumvoll (NRI = 0.603), and Cederholm (NRI = 0.601) were the best indexes for reclassifying T2D risk, while the TG/G (NRI = 0.068) was the worst.

**Table 4:** Cox hazard and AUC models in the population study for the associations of surrogate indexes with risk and prediction of type 2 diabetes development

Index	Population study					
	n = 2260					
	HR (95% CI)	*P*	AUC	*P*-value for AUC comparison with Cederholm's AUC	*P*-value for AUC comparison with HOMA-IR's AUC	NRI
Requiring an OGTT						
Gutt	2.34 (2.07-2.64)	<.0001	0.728	.519	.0070	0.567
Matsuda	2.11 (1.89-2.34)	<.0001	0.715	.0404	.0129	0.613
Belfiore	1.98 (1.79-2.20)	<.0001	0.707	<.0001	.5190	0.532
Avignon (Si2h)	1.75 (1.57-1.94)	<.0001	0.683	<.0001	.1146	0.447
Avignon (SiM)	1.88 (1.69-2.11)	<.0001	0.692	<.0001	.2594	0.500
Avignon (Sib)	1.76 (1.59-1.95)	<.0001	0.683	<.0001	<.0001	0.509
Stumvoll	1.45 (1.38-1.52)	<.0001	0.705	.0020	.6302	0.603
Cederholm	2.28 (2.04-2.55)	<.0001	0.728	—	.0130	0.601
Requiring fasting insulin						
HOMA-IR	1.97 (1.77-2.19)	<.0001	0.701	.0130	—	0.577
QUICKI	2.04 (1.81-2.28)	<.0001	0.701	.0134	.6921	0.542
McAuley	1.69 (1.53-1.86)	<.0001	0.682	.0001	.0037	0.477
Fasting insulin	1.84 (1.66-2.04)	<.0001	0.687	.0002	<.0001	0.522
Not requiring insulin measures						
SPISE	1.77 (1.61-1.94)	<.0001	0.682	.0007	.0865	0.456
METS-IR	1.80 (1.64-1.98)	<.0001	0.688	.0027	.2184	0.502
Lipid accumulation product	1.47 (1.35-1.61)	<.0001	0.658	<.0001	.0004	0.343
Triglycerides and HDL cholesterol ratio	1.28 (1.17-1.40)	<.0001	0.630	<.0001	<.0001	0.267
Triglycerides and glucose index	1.33 (1.21-1.45)	<.0001	0.637	<.0001	<.0001	0.340
Triglycerides and glucose ratio	1.06 (0.97-1.16)	.1957	0.605	<.0001	<.0001	0.068

Associations of each index with the risk of diabetes were studied separately. All models were adjusted for age, sex, and fraction of American Indian heritage.

All indexes were log-transformed and then standardized. HRs are given per 1 SD of the log-transformed index for indexes measuring insulin resistance; for indexes measuring insulin sensitivity, the HRs and 95% CIs shown are 1/estimated HR (1/upper limit of 95% CI, 1/lower limit of 95% CI).

The AUCs for the baseline models that only included the covariates were population study = 0.604.

The NRIs were calculated at the median follow-up time.

— indicates measure not available or comparison not performed.

Abbreviations: AUC, area under the receiver operating curve; CI, confidence interval; HOMA-IR, Homeostatic Model Assessment of Insulin Resistance; HDL, high-density lipoprotein; HR, hazard ratio; METS-IR, metabolic score for insulin resistance; NRI, net reclassification improvement; OGGT, oral glucose tolerance test; QUICKI, quantitative insulin sensitivity check index; SPISE, single-point insulin sensitivity index.

When these analyses were conducted with additional adjustment for BMI, results were generally similar to those conducted without including BMI in the models (Table 5). NRIs were generally attenuated, particularly for indexes that include BMI in their calculation. Cederholm had significantly higher AUC than all other indexes, except Gutt, and HOMA-IR had significantly higher AUC than all indexes that do not include insulin measurements. Additional adjustment for HbA1c and current smoking and drinking resulted in modest further attenuation but produced similar results (Table S4) (41).

**Table 5:** Cox hazard and AUC models in the population study for the associations of surrogate indexes with risk and prediction of type 2 diabetes development, with adjustment for BMI

Index	Population study n = 2260					
	HR (95% CI)	*P*	AUC	*P*-value for AUC comparison with Cederholm's AUC	*P*-value for AUC comparison with HOMA-IR's AUC	NRI
Requiring an OGTT						
Gutt	2.12 (1.87-2.41)	<.0001	0.737	.8625	.0005	0.543
Matsuda	1.89 (1.68-2.12)	<.0001	0.721	.0017	.0062	0.544
Belfiore	1.80 (1.61-2.00)	<.0001	0.719	<.0001	.1451	0.485
Avignon (Si2h)	1.71 (1.54-1.91)	<.0001	0.716	<.0001	.4177	0.436
Avignon (SiM)	1.77 (1.58-1.99)	<.0001	0.716	<.0001	.2500	0.468
Avignon (Sib)	1.62 (1.46-1.80)	<.0001	0.709	.0012	.9323	0.456
Stumvoll	1.37 (1.30-1.44)	<.0001	0.716	.0016	.1965	0.555
Cederholm	2.07 (1.84-2.33)	<.0001	0.737	—	.0012	0.529
Requiring fasting insulin						
HOMA-IR	1.73 (1.54-1.95)	<.0001	0.709	.0012	—	0.483
QUICKI	1.76 (1.55-2.00)	<.0001	0.708	.0010	.3022	0.492
McAuley	1.49 (1.35-1.66)	<.0001	0.697	<.0001	.0116	0.340
Fasting insulin	1.59 (1.41-1.78)	<.0001	0.699	<.0001	<.0001	0.363
Not requiring insulin measures						
SPISE	1.90 (1.52-2.39)	<.0001	0.682	<.0001	.0022	0.246
METS_IR	2.35 (1.85-2.99)/	<.0001	0.689	<.0001	.0227	0.296
Lipid accumulation product	1.22 (1.09-1.36)	.0003	0.678	<.0001	.0005	0.222
Triglycerides and HDL cholesterol ratio	1.23 (1.12-1.35)	<.0001	0.680	<.0001	.0012	0.249
Triglycerides and glucose index	1.28 (1.17-1.41)	<.0001	0.686	<.0001	.0100	0.313
Triglycerides and glucose ratio	1.05 (0.96-1.15)	.2838	0.675	<.0001	.0001	0.089

Associations of each index with the risk of diabetes were studied separately. All models were adjusted for age, sex, body mass index, and fraction of American Indian heritage.

All indexes were log-transformed and then standardized. HRs are given per 1 SD of the log-transformed index for indexes measuring insulin resistance; for indexes measuring insulin sensitivity, the HRs and 95% CIs shown are 1/estimated HR (1/upper limit of 95% CI, 1/lower limit of 95% CI).

The AUCs for the baseline models that only included the covariates were: population study = 0.675.

The NRIs were calculated at the median follow-up time.

— indicates measure not available or comparison not performed.

Abbreviations: AUC, area under the receiver operating curve; CI, confidence interval; HOMA-IR, Homeostatic Model Assessment of Insulin Resistance; HDL, high-density lipoprotein; HR, hazard ratio; METS-IR, metabolic score for insulin resistance; NRI, net reclassification improvement; OGGT, oral glucose tolerance test; QUICKI, quantitative insulin sensitivity check index; SPISE, single-point insulin sensitivity index.

In the sensitivity analysis cohort, HRs for indexes based on OGTTs remained higher than those for indexes that were not based on OGTTs, as did most AUCs (Table S5) (41). HRs and AUCs were generally higher than in the population study cohort. Gutt's HR increased to 2.69 and was the strongest association with T2D risk. Cederholm still had a significantly higher AUC (0.750) than most other indexes. Indexes requiring OGTT generally performed better according to NRI calculations than indexes that do not use insulin measures when predicting T2D.

### Diabetes in the HEC Substudy Cohort

In this cohort, the gold-standard measure of insulin sensitivity, M (HR 2.56, 95% CI 1.42-4.60), had the strongest association with T2D development (Table S6) (41). In this smaller sample, indexes based on fasting insulin were more weakly associated with the risk of diabetes development. Stumvoll (AUC = 0.751) and Cederholm (AUC = 0.741) indexes had the highest AUCs for predicting future T2D in the HEC substudy, and these were significantly higher than the AUC for M (0.673). The strongest index for reclassifying risk of T2D according to NRI values was Stumvoll.

## Discussion

Insulin resistance is a key component of glucose homeostasis and an important factor in the development of diabetes. Therefore, assessment of insulin resistance to glucose disposal is important to determine the risk of developing T2D. The gold-standard methods for measuring insulin resistance, such as the HEC, are too expensive and labor-intensive for implementation in large-scale epidemiologic studies and clinical situations. Measures based on the frequently sampled IV glucose tolerance test [eg, the “minimal model” (42)] are less labor-intensive but still require specially trained staff. Thus, many investigators have developed surrogate measures of insulin resistance/sensitivity that are more convenient. Some of these indexes require measurements of insulin and glucose from OGTTs, some require measurements of fasting insulin concentrations without an oral glucose load, while others are based on biomarkers available in routine clinical care such as fasting serum triglycerides and glucose. Several extensions of the minimal model that use data from the OGTT have been developed, but these generally require samples at multiple time points, and, thus, they are not clinically convenient (43, 44). In the present study, we evaluate several indexes of insulin resistance in a cohort of indigenous Americans living in the southwest United States. We estimated their correlations with the gold-standard measures of insulin resistance derived from the HEC and investigated their ability to predict development of T2D.

In this study, HECs were performed on a subset of individuals, which allowed us to compare surrogate indexes to the gold-standard measure of insulin resistance/sensitivity, M. The Matsuda index had the strongest correlation with M compared to all others, and its correlation with M was statistically stronger than that for any of the other indexes. Our results are consistent with those of a meta-analysis by Otten et al (45) that concluded that Matsuda correlated best with insulin sensitivity measures from the HEC, though statistical significance among correlations was not assessed. Lorenzo et al (46) also found that Matsuda's index was significantly more strongly correlated with M than most other indexes. Both studies found that QUICKI and HOMA-IR (which are highly correlated with one another) provided the strongest correlations with M among surrogate indexes based on fasting insulin concentrations, and our findings are consistent with this. In fact, HOMA-IR/QUICKI correlations with M were nearly as strong as those for Matsuda, and this suggests that HOMA-IR/QUICKI can provide suitable information about insulin resistance when OGTT data are not available. Among measures that do not require insulin measurements, SPISE and METS-IR had the strongest correlations with M, but the information about insulin resistance is less than for the indexes based on OGTTs or fasting insulin.

Although the correlations with M of most of the indexes examined were highly statistically significant, the magnitude of the correlations was modest to moderate. The sensitivity of these indexes for detecting insulin resistance, defined by the lowest quartile of M, was also modest to moderate. Thus, there is some degree of misclassification, relative to the clamp measure, when using the indexes, even those based on the OGTT, to diagnose insulin resistance. As in many epidemiologic studies, the present analyses were based on a single measurement of the indexes, and the use of repeated measures may improve clinical utility.

In assessing the risk of diabetes in the HEC substudy cohort, the gold standard, M, had 1 of the stronger associations to predict the development of T2D as measured by the HR. However, in this small cohort, several surrogate indexes had significantly higher AUCs than M for the prediction of future T2D. Given the variability in this small cohort, conclusions about the relative value for predicting T2D are best made from results in the larger population cohort, where statistical power is much greater.

Results from this large-scale population cohort show that OGTT-requiring indexes were best in assessing the risk and predicting diabetes. Among indexes requiring measurement of fasting insulin but not OGTT data, HOMA-IR/QUICKI were the strongest predictors of T2D. These findings suggest that, when OGTT data are not available, HOMA-IR and QUICKI provide optimal assessment of insulin resistance and diabetes risk. These findings are concordant with those from other cohorts (47, 48). In the present study, the Cederholm and Gutt indexes had the highest NRI and AUC values, and the AUC was significantly higher than that of all other indexes. These results are in accordance with other prospective studies in a multiethnic cohort (47) and, most recently, a Japanese American cohort (48), where the Gutt index was the strongest predictor of T2D. In the present study, Gutt was a slightly but significantly stronger predictor of diabetes than Matsuda, despite the lower correlation with M. This suggests that perhaps Gutt also captures other risk factors for T2D, such as insulin secretion. In fact, we found that, with adjustment for M, Gutt and Cederholm were also associated with diminished insulin secretion, which is a strong risk factor for diabetes in this population (49). Thus, differences among indexes in the ability to predict diabetes may reflect other aspects of insulin-glucose metabolism in addition to whole body insulin resistance.

Although indexes not requiring insulin measures did not perform as strongly as the other surrogate indexes, they still may provide useful information, especially when insulin measures are unavailable or cost is an issue. In our cohort, SPISE and METS-IR had the highest HR for assessing the risk of diabetes among indexes not utilizing insulin in their calculations, while the METS-IR index had the highest AUC and NRI. The AUC for other measures that do not utilize insulin in their calculation, including TyG, was significantly lower than that for HOMA-IR. These results are discordant with those from Park et al (50) who studied a Korean cohort, in which the TyG index was better at predicting T2D than HOMA-IR. SPISE and METS-IR had a significantly stronger correlation with M than any of the other indexes not requiring insulin measures. Our results suggest that either SPISE or METS-IR may be useful surrogate measures in situations where measurement of serum insulin is impractical. On the other hand, the triglyceride/glucose ratio and triglyceride/HDL ratio had weak correlations with M and weak associations with the development of T2D, and these are likely of limited clinical utility. The lack of standardization of insulin assays limits the ability to compare surrogate measures based on insulin measurements across different cohorts, and this remains a barrier to widespread clinical use of these indexes (51).

Obesity is strongly related to insulin resistance, and in some clinical and research situations, it may be useful to assess the effects of insulin resistance that are independent of obesity. In the present study, adjustment for BMI attenuated the predictive properties of most indexes, but the relative utility was similar to that from analyses without this adjustment. Further adjustment for HbA1c, current smoking, and current use of alcohol produced similar results, with most surrogate indexes showing highly statistically significant associations with incident T2D. These analyses suggest that assessment of surrogate measures of insulin resistance, especially when insulin levels are measured, provide substantial information about T2D risk beyond that provided by commonly measured clinical factors.

An advantage of the present study is the relatively large sample with many follow-up years for the prediction of diabetes. Other strengths include the collection of all measures in a uniform and standardized manner, as well as all testing being conducted in the same laboratory throughout the entire study. There are some limitations. The use of the 2-hour glucose level to diagnose diabetes in the study may have favored indexes based on the OGTT. However, our sensitivity analysis found similar results when diagnoses were based on fasting glucose and HbA1c but not 2-hour glucose. Our study population of indigenous Americans has a high prevalence of diabetes; therefore, the findings in this study may not be applicable to other populations. We limited analyses to indexes that can be easily calculated using measurements that are currently readily available clinically. In the future, novel approaches, such as machine learning and metabolomics, may provide a better assessment of insulin resistance (52).

In summary, surrogate measures of insulin resistance can be useful for the assessment of insulin sensitivity and the prediction of diabetes risk in situations where more intensive methods, such as the HEC, are not practical. Most indexes evaluated in our analysis have some utility for the prediction of future T2D. In situations where measurement of serum insulin is not practical, SPISE and METS-IR can be of clinical utility. However, indexes that use serum insulin measures tend to provide more information than those that do not use insulin measures, and indexes that use glucose and insulin levels during an OGTT provide more information than those based on fasting measures alone.

## Data Availability

Restrictions apply to the availability of some or all data generated or analyzed during this study to preserve patient confidentiality or because they were used under license. The corresponding author will on request detail the restrictions and any conditions under which access to some data may be provided.

## Prior presentation

This work was presented in part at the 85th Scientific Sessions of the American Diabetes Association, Chicago, Illinois, June 20-23, 2025.
